# Facial Angioedema after the first dose of Covishield (adenovirus-vectored severe acute respiratory syndrome coronavirus 2 vaccine): follow-up after the second and third booster doses

**DOI:** 10.1590/0037-8682-0063-2022

**Published:** 2022-05-20

**Authors:** Walter de Araujo Eyer-Silva, Lidiane Simões de Carvalho Paes Leme

**Affiliations:** 1 Universidade Federal do Estado do Rio de Janeiro, Hospital Universitário Gaffrée e Guinle, Rio de Janeiro, RJ, Brasil.

**Keywords:** Angioedema, COVID-19, Covishield

## Abstract

Mass vaccination campaigns are essential to control the ongoing novel severe acute respiratory syndrome coronavirus 2 (SARS-Cov-2) pandemic. The Covishield vaccine consists of the replication-deficient simian adenovirus vector ChAdOx1, which contains the full-length structural spike protein of SARS-CoV-2. Occasionally, it can lead to cutaneous reactions that contribute to fear of vaccination, hesitancy, and incomplete vaccination schedules. We report a case of facial angioedema following the first dose of Covishield in a 63-year-old woman with no previous history of allergies or hypersensitivity to drugs or vaccines. No rebound of angioedema was recorded after the second homologous and third heterologous doses.

## INTRODUCTION

Mass vaccination campaigns are essential to control the ongoing novel severe acute respiratory syndrome coronavirus 2 (SARS-Cov-2) pandemic. On January 17, 2021, the Brazilian Health Regulatory Agency authorized emergency use in Covishield, the Oxford-AstraZeneca nCoV-19 vaccine^1^, which consists of the replication-deficient simian adenovirus vector ChAdOx1, containing the full-length structural surface glycoprotein (spike protein) of SARS-CoV-2^2^. This vaccine has an excellent safety profile, although it has been linked to rare cases of vaccine-induced immune thrombosis and thrombocytopenia^3^. Covishield and other SARS-Cov-2 vaccines may also lead to cutaneous reactions, mainly at local sites, and delayed large local reactions. Urticaria, vasculopathic manifestations, pityriasis rosea-like eruptions, and herpes zoster have also been associated with Covishield and other SARS-Cov-2 vaccines^4^ ^)-(^ ^5^. We report a case of facial angioedema that followed the first dose of Covishield. No rebound of angioedema was recorded after the second homologous and third heterologous doses.

## CASE REPORT

**FIGURE 1:**
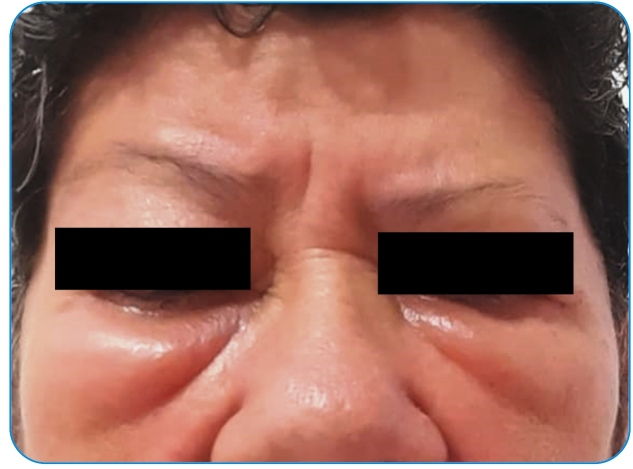
Clinical image of a 63-year-old woman showing bilateral conjunctival angioedema after the first dose of the adenovirus-vectored (ChAdOx1) SARS-Cov-2 vaccine. No reactions were recorded after the second homologous and third heterologous booster doses.

A 63-year-old woman with no history of allergies or hypersensitivity to drugs or vaccines presented for SARS-Cov-2 immunization at a medical facility in Rio de Janeiro. She regularly used antihypertensive medications and had no other significant medical history. At 9 AM, the first shot of Covishield was administered. Nine hours later, she first noticed the emergence of bilateral conjunctival and facial angioedema (Figure 1) accompanied by local pruritus and a burning sensation. As the angioedema did not remit spontaneously in the following hours, she sought medical advice and was treated with parenteral administration of promethazine and hydrocortisone. Complete remission was achieved within a few hours. Between the first and second doses of the vaccine schedule, she moved to another state of the federation. Due to the history of the previous reaction, local health personnel was reluctant to offer a second dose of Covishield, which was only administered 106 days after the first shot. Instead, a second-generation antihistamine was used as premedication. Subsequently, a Pfizer-BioNTech booster dose was administered 141 days after the second dose without premedication. No hypersensitivity reactions were observed after the second or booster doses.

## DISCUSSION

Cutaneous reactions to SARS-Cov-2 vaccines may lead to fear, hesitation, and incomplete vaccination schedules. Most of these reactions are benign, and patients may be safely reassured to complete their immunization schedule. Such cases must be reported to fully understand the clinical spectrum and epidemiology of SARS-Cov-2 vaccine reactions. Our patient presented with angioedema a few hours after receiving the first Covishield dose. The fear of a novel reaction led to a substantial delay in the administration of the second dose. However, the patient eventually tolerated both homologous and heterologous booster doses. Although angioedema is considered a typical manifestation of immediate-type reactions, it can also occur with delayed reactions^6^. Angioedema has been reported after SARS-Cov-2 vaccines^7^ ^)-(^ ^8^, including Covishield, but the underlying pathophysiological mechanism remains unknown. It is important to note that angioedema has also been described as a manifestation of SARS-Cov-2 infection itself^9^ ^)-(^ ^10^.
